# Timing of Cefuroxime Surgical Antimicrobial Prophylaxis and Its Association With Surgical Site Infections

**DOI:** 10.1001/jamanetworkopen.2023.17370

**Published:** 2023-06-08

**Authors:** Rami Sommerstein, Nicolas Troillet, Stephan Harbarth, Marlieke E.A. de Kraker, Danielle Vuichard-Gysin, Stefan P. Kuster, Andreas F. Widmer

**Affiliations:** 1 Swissnoso, the National Center for Infection Control, Bern, Switzerland; 2 Department of Infectious Diseases, Bern University Hospital, University of Bern, Bern, Switzerland; 3 Department of Health Sciences and Medicine, University of Lucerne, Lucerne, Switzerland; 4 Service of Infectious Diseases, Central Institute, Valais Hospitals, Sion, Switzerland; 5 Infection Control Program, Geneva University Hospitals and Faculty of Medicine, World Health Organization Collaborating Center, Geneva, Switzerland; 6 Division of Infectious Diseases and Hospital Epidemiology, Cantonal Hospital Thurgau, Muensterlingen and Frauenfeld, Switzerland; 7 Division of Infectious Diseases and Hospital Epidemiology, Cantonal Hospital St Gallen, St Gallen, Switzerland; 8 Department of Infectious Diseases, University Hospital Basel, Basel, Switzerland

## Abstract

**Importance:**

World Health Organization guidelines recommend administering surgical antimicrobial prophylaxis (SAP), including cefuroxime, within 120 minutes prior to incision. However, data from clinical settings supporting this long interval is limited.

**Objective:**

To assess whether earlier vs later timing of administration of cefuroxime SAP is associated with the occurrence of surgical site infections (SSI).

**Design, Setting, and Participants:**

This cohort study included adult patients who underwent 1 of 11 major surgical procedures with cefuroxime SAP, documented by the Swissnoso SSI surveillance system between January 2009 and December 2020 at 158 Swiss hospitals. Data were analyzed from January 2021 to April 2023.

**Exposures:**

Timing of cefuroxime SAP administration before incision was divided into 3 groups: 61 to 120 minutes before incision, 31 to 60 minutes before incision, and 0 to 30 minutes before incision. In addition, a subgroup analysis was performed with time windows of 30 to 55 minutes and 10 to 25 minutes as a surrogate marker for administration in the preoperating room vs in the operating room, respectively. The timing of SAP administration was defined as the start of the infusion obtained from the anesthesia protocol.

**Main Outcomes and Measures:**

Occurrence of SSI according to Centers for Disease Control and Prevention definitions. Mixed-effects logistic regression models adjusted for institutional, patient, and perioperative variables were applied.

**Results:**

Of 538 967 surveilled patients, 222 439 (104 047 men [46.8%]; median [IQR] age, 65.7 [53.9-74.2] years), fulfilled inclusion criteria. SSI was identified in 5355 patients (2.4%). Cefuroxime SAP was administered 61 to 120 minutes prior to incision in 27 207 patients (12.2%), 31 to 60 minutes prior to incision in 118 004 patients (53.1%), and 0 to 30 minutes prior to incision in 77 228 patients (34.7%). SAP administration at 0 to 30 minutes was significantly associated with a lower SSI rate (adjusted odds ratio [aOR], 0.85; 95% CI, 0.78-0.93; *P* < .001), as was SAP administration 31 to 60 minutes prior to incision (aOR, 0.91; 95% CI, 0.84-0.98; *P* = .01) compared with administration 61 to 120 minutes prior to incision. Administration 10 to 25 minutes prior to incision in 45 448 patients (20.4%) was significantly associated with a lower SSI rate (aOR, 0.89; 95% CI, 0.82-0.97; *P* = .009) vs administration within 30 to 55 minutes prior to incision in 117 348 patients (52.8%).

**Conclusions and Relevance:**

In this cohort study, administration of cefuroxime SAP closer to the incision time was associated with significantly lower odds of SSI, suggesting that cefuroxime SAP should be administrated within 60 minutes prior to incision, and ideally within 10 to 25 minutes.

## Key Points

**Question:**

What is the optimal timing of cefuroxime surgical antimicrobial prophylaxis?

**Findings:**

In this cohort study of 222 439 patients who underwent 1 of 11 major surgical procedures, administration of cefuroxime surgical antimicrobial prophylaxis closer to the time of incision, vs earlier administration, was associated with significantly lower odds of surgical site infection.

**Meaning:**

These findings suggest that cefuroxime surgical prophylaxis should be administrated within 60 minutes prior to incision, ideally within 10 to 25 minutes.

## Teaser

This cohort study assesses whether the timing of the administration of cefuroxime surgical antimicrobial prophylaxis prior to incision is associated with the occurrence of surgical site infections.

## Introduction

Surgical site infections (SSIs) account for approximately 20% of all health care–associated infections^1,2^ and have a major impact on morbidity and mortality.^3,4^ Several national and international guidelines provide evidence-based measures to prevent SSI. Several factors related to surgical antimicrobial prophylaxis (SAP), including choice of SAP, timing, and redosing, have been identified as crucial for SSI prevention.^4,5,6,7,8^

The association of the timing of SAP administration with risk of SSI has been described in early experimental animal studies.^9^ The landmark study by Classen and colleagues^10^ in 1992 showed that the lowest risk of SSI in human beings was when SAP was administered within 2 hours of skin incision. A relevant shortcoming of this study was the heterogeneity in antibiotics and the prolonged dosing, sometimes well beyond 24 hours. The 2016 World Health Organization guidelines^4^ for the prevention of SSI call for timing the administration of SAP to be less than 120 minutes before incision and conclude that, on the basis of the available evidence, it is not possible to establish the optimal timing more precisely within the 120-minute interval. Because of concerns that serum and tissue concentrations of antibiotics with a short half-life (such as cefazoline and cefuroxime) may be less effective if given too early in this time interval, several international guidelines^8,11,12^ suggest initiating SAP within 60 minutes before incision.

However, the optimal time to initiate SAP within the 60-minute window is debated. A large prospective cohort study^13^ on cefuroxime SAP suggested that administration of SAP closer to the incision time might be too late for optimal SSI prevention. In contrast, a 2017 randomized superiority trial^14^ did not find a difference between SAP administered approximately 30 to 55 minutes vs 10 to 25 minutes before incision. The findings of the study^14^ did not support any narrowing of the 60-minute window for the administration of a cephalosporin with a short half-life.

SAP administration immediately upon entering the operating room could also be beneficial; a before-after study^15^ suggested that the number of patients without completion of SAP prior to surgical incision decreased significantly from 16.8% to 1.8% if SAP was administered immediately after the patients entered the operating room. The aim of this cohort study was, therefore, to assess whether the timing of administration (ie, at 61-120 minutes vs at 31-60 minutes vs at 0-30 minutes) of cefuroxime SAP was associated with different rates of SSI, and, in addition, whether administration of SAP after the patient is transferred to the operating room (10-25 minutes before incision) is an optimal administration time.

## Methods

This study is based on data from the Swissnoso SSI surveillance system,^16,17^ which is mandated by the Swiss National Association for Quality Development in Hospitals and Clinics. All patients were informed about their automatic inclusion in SSI surveillance at admission and were given the opportunity to opt out. Because the Swissnoso SSI surveillance system is a quality improvement project, no individual patient consent was needed, but the Bernese Cantonal human subjects committee approved risk factors analyses within the SSI surveillance database. Summary results of the SSI incidences are published yearly.^18^ This cohort study follows theStrengthening the Reporting of Observational Studies in Epidemiology (STROBE) reporting guideline.^19^

### Study Design and Setting

This is a retrospective multicenter cohort study of prospectively collected data from the Swiss national SSI surveillance program (established in 2009), which currently has 168 participating centers.^16,17^ We included data from 158 health care institutions in Switzerland that reported data with at least 1 eligible patient between January 2009 and December 2020. A total of 10 centers did not use cefuroxime as SAP and were excluded. Each participating hospital records surveillance data on a minimum of 3 different procedure types reporting on all patients during a preselected period.^16^ The surveillance includes data collection of baseline characteristics, surgical procedure, and outcomes at discharge, as well as postdischarge (active follow-up 30 days after the procedure and at 1 year for arthroplasty operations), with additional medical record review in case of suspected infection.^16^ Surveillance time frames did not change throughout the study period. Time of SAP administration was taken from the anesthesia protocol and corresponded to the start of the antibiotic infusion. All patients were contacted at least 5 times before being considered lost to follow-up. The overall follow-up for routine postdischarge surveillance was greater than 91%.^16^

Surveillance staff reviewed all patient data, and patients with a suspected SSI were validated by a dedicated physician. Staff members of the Swissnoso SSI surveillance team periodically performed on-site audits to check data quality, which are published elsewhere.^16,17,18,20,21^

### Participants

**Figure 1.**
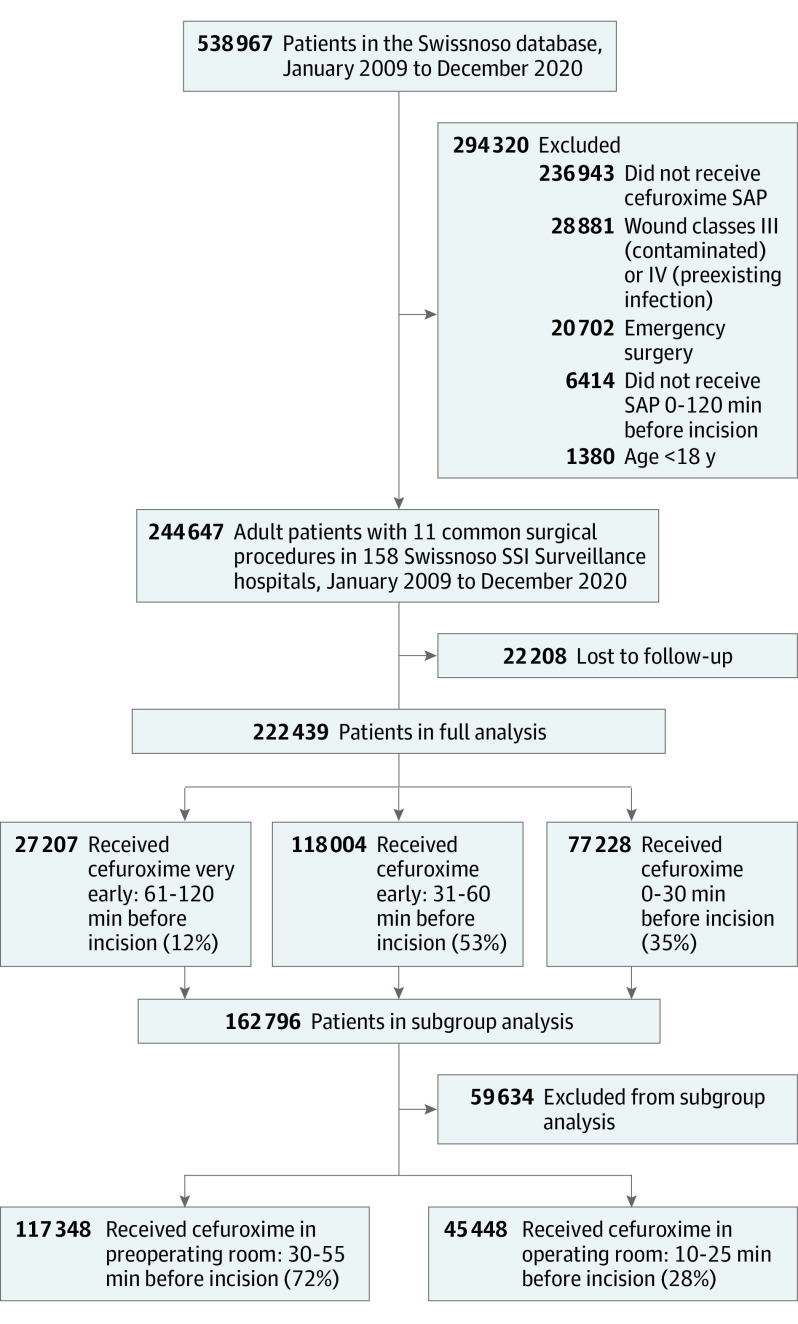
Participant Flowchart

Inclusion criteria were (1) participation in the surveillance program, (2) undergoing 1 of the 11 most frequent surgical interventions (hernia repair, knee arthroplasty, hip arthroplasty, cardiac surgery, laminectomy, spondylodesis, colon surgery, cholecystectomy, cesarean delivery, gastric bypass, or hysterectomy), (3) the procedure taking place between 2009 and 2020, (4) being older than 18 years, and (5) a cefuroxime (with or without metronidazole) SAP administration in the 120 minutes before incision. Exclusion criteria were patients with a class III or IV wound contamination (ie, preexisting infection), patients who had emergency surgery, and patients with incomplete follow-up (Figure 1).

### Variables, Outcomes, and Data Sources

The primary outcome was any SSI (superficial or deep incisional infection and/or organ space infection) at 30 days and/or 1 year. Covariables included age; American Society of Anesthesiologists (ASA) score, recorded according to the 1961 definitions^22^; wound contamination class I (clean) or class II (clean-contaminated) according to Culver et al^23^; year of surgery; hospital bed size; procedure type; and procedure duration longer than standard time. The 75th percentile of surgery time is referred to as the procedure-specific T-time; the T-times are taken from the National Nosocomial Infections Surveillance System surveillance report.^23^ The decision for the categorical SAP timing groups (61-120 minutes vs 31-60minutes vs 0-30 minutes) was planned a priori and was based on the results and methods of previous cohort studies,^13,24^ whereas the timing windows for the subgroup 30 to 55 minutes (preparatory room administration as proxy for application in the preoperating room) vs 10 to 25 minutes (as proxy for administration in the operating room) were based on the IQRs of SAP administration timing in a previous randomized clinical trial (RCT).^14^ The main exposure, SAP administration (including SAP choice and rate of administration), depended on institution-specific guidelines, but timing of SAP administration was always defined as the start of the infusion.

SSI cases were defined as patients with SSI according to US Centers for Disease Control and Prevention definitions.^25^ Type of SSI (ie, superficial incision, deep incisional, or organ space) was recorded, as well as the microbial cause (if available). Data were electronically entered into a centralized database.

To analyze the consequences of preoperative comorbidity, ASA scores were grouped into low^1,2^ and high scores.^3,4,5^ Age was grouped into younger than 40 years and 40 years or older. Regarding bed size, hospitals were grouped into those with fewer than 200 beds, 200 to 500 beds, and more than 500 beds.

### Statistical Analysis

To investigate differences in terms of baseline characteristics within the 3 SAP timing groups, we used the χ^2^ and Wilcoxon tests for categorical and continuous data, respectively. The SSI outcome per type of surgical intervention was also calculated by the SAP timing groups. To determine the association of SAP timing with SSI, we fitted multilevel logistic regression models with clustering at the procedure level (random intercept), adjusted for all covariates.

A 2-tailed *P* < .05 was considered statistically significant throughout. All statistics were performed using R statistical software version 4.0.2 (R Project for Statistical Computing).^26^ Data analysis was conducted from January 2021 to April 2023.

## Results

Of 538 967 patients in the database, 244 647 patients (45.4%) were eligible, of whom 22 208 (9.1%) were lost to follow-up (Figure 1). Characteristics of included patients and those lost-to follow-up are compared in eTable 1 in Supplement 1. In total, 222 439 patients (104 047 men [46.8%]; median [IQR] age, 65.7 [53.9-74.2] years) were included. Cefuroxime SAP was administered at a median (IQR) of 38 (25-60) minutes before incision. SAP was administered 61 to 120 minutes before incision in 27 207 patients (12.2%), 31 to 60 minutes before incision in 118 004 patients (53.1%), and 0 to 30 minutes before incision in 77 228 patients (34.7%) (Figure 1). A histogram of SAP administration relative to incision is shown in the eFigure in Supplement 1. The detailed baseline patient and procedural characteristics stratified by SAP timing are shown in Table 1. Of importance, over 50% of patients (123 174 patients; 55.4%) underwent an arthroplasty procedure. Older patients, those with higher ASA scores, those with class II clean-contaminated wounds, those receiving care at larger hospitals, and those with more complex surgery types were more likely to be in the earlier SAP timing group (ie, 61-120 minutes) (Table 1). We only had information on the cefuroxime dosing regimen for 52.0% of the cohort (115 761participants) and information on body-mass index for 64.0% of the cohort (140 232 participants), therefore these 2 variables were excluded from analysis.

**Table 1.** Baseline and Procedural Characteristics of Patients by Timing of SAP Administration

Characteristic	Patients, No (%) (N = 222 439)			*P *value
	0-30 min (n = 77 228)	31-60 min (n = 118 004)	61-120 min (n = 27 207)	
Age, median (IQR), y	62.41 (45.74-72.78)	66.93 (56.86-74.75)	67.25 (57.19-75.05)	<.001
Sex				
Female	44 175 (57.2)	60 775 (51.5)	13 442 (49.4)	<.001
Male	33 053 (42.8)	57 229 (48.5)	13 765 (50.6)	
American Society of Anesthesiologists score				
1-2	57 719 (74.7)	79 688 (67.5)	17 342 (63.7)	<.001
3-5	19 094 (24.7)	37 938 (32.1)	9733 (35.8)	
NA	415 (0.5)	378 (0.3)	132 (0.5)	
Addition of metronidazole as second SAP	6255 (6.8)	10 080 (8.5)	4062 (14.9)	<.001
Procedure type				
Cesarean delivery	10 375 (13.4)	2242 (1.9)	809 (3.0)	<.001
Cholecystectomy	5241 (6.8)	4021 (3.4)	569 (2.1)	
Colon surgery	4287 (5.6)	8995 (7.6)	3935 (14.5)	
Hernia repair	12 275 (15.9)	7235 (6.1)	1142 (4.2)	
Hysterectomy	2916 (3.8)	2385 (2.0)	225 (0.8)	
Cardiac surgery	3372 (4.4)	10 492 (8.9)	3468 (12.7)	
Laminectomy	2064 (2.7)	3772 (3.2)	750 (2.8)	
Spondylodesis	627 (0.8)	1636 (1.4)	522 (1.9)	
Gastric bypass surgery	2283 (3.0)	2993 (2.5)	634 (2.3)	
Total hip arthroplasty	19 903 (25.8)	43 465 (36.8)	8164 (30.0)	
Total knee arthroplasty	13 885 (18.0)	30 768 (26.1)	6989 (25.7)	
Wound contamination class II (clean-contaminated)	25 391 (32.9)	21 080 (17.9)	6261 (23.0)	<.001
Surgery exceeded standard time	8801 (11.4)	17 160 (14.5)	5846 (21.5)	<.001
Year of procedure, median (IQR)	2015 (2013-2018)	2016 (2013-2018)	2015 (2013-2017)	<.001
Hospital bed size, No. of beds				
<200	44 510 (57.6)	67 150 (56.9)	15 287 (56.2)	<.001
200-500	25 021 (32.4)	34 888 (29.6)	7607 (28.0)	
>500	7697 (10.0)	15 966 (13.5)	4313 (15.9)	

Abbreviations: NA, not available; SAP, surgical antimicrobial prophylaxis.

**Figure 2.**
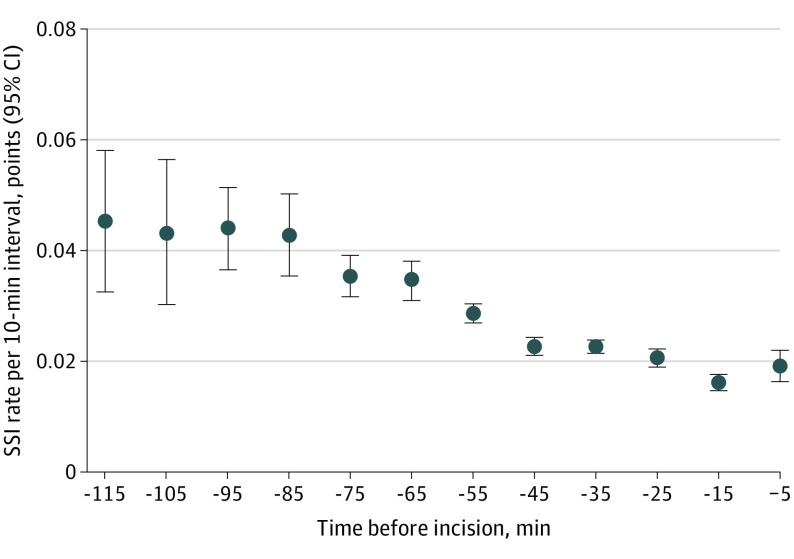
Crude Surgical Site Infection (SSI) Rate Relative to Timing of Surgical Antimicrobial Prophylaxis (SAP) Graph shows crude SSI rate relative to timing of SAP administration per 10-minute timing window. For example, −15 minutes stands for SAP administration in the time window −19 to −10 minutes prior to incision. Bars denote 95% CIs, and dots denote mean SSI rate.

We plotted the crude SSI rate relative to timing of SAP in Figure 2. The overall rate of SSI was 2.4% (5355 patients), with an SSI rate of 1.9% (1468 patients) in the 0 to 30 minute timing group, 2.4% (2873 patients) in the 31 to 60 minute timing group, and 3.7% (1013 patients) in the 61 to 120 minute timing group.

The crude SSI rates between the 3 timing groups, stratified for the surgical procedures, indicated a higher SSI rate with early SAP administration (ie, 61-120 minutes before incision) for cesarean delivery, cholecystectomy, colon surgery, and gastric bypass compared with administration just prior to incision (Table 2). The summary of the leading microorganism detected in 3381 of 222 439 cases (1.5%) with recorded cause by SAP timing group is shown in eTable 2 in Supplement 1.

**Table 2.** Crude SSI Rate per SAP Timing Group and Procedure Type

Procedure type and infections	Patients, No. (%)		
	0-30 min	31-60 min	61-120 min
Cesarean delivery			
Patients, No.	10 375	2242	809
Overall infection	115 (1.1)	34 (1.5)	13 (1.6)
Superficial incisional infection	71 (0.7)	21 (0.9)	9 (1.1)
Deep incisional infection	13 (0.1)	2 (0.1)	1 (0.1)
Organ incisional infection	31 (0.3)	11 (0.5)	3 (0.4)
Cholecystectomy			
Patients, No.	5241	4021	569
Overall infection	62 (1.2)	60 (1.5)	15 (2.6)
Superficial incisional infection	36 (0.7)	27 (0.7)	9 (1.6)
Deep incisional infection	3 (0.1)	8 (0.2)	3 (0.5)
Organ incisional infection	23 (0.4)	25 (0.6)	3 (0.5)
Colon surgery			
Patients, No.	4287	8995	3935
Overall infection	448 (10.5)	988 (11.0)	482 (12.2)
Superficial incisional infection	125 (2.9)	279 (3.1)	144 (3.7)
Deep incisional infection	52 (1.2)	97 (1.1)	52 (1.3)
Organ incisional infection	271 (6.3)	612 (6.8)	286 (7.3)
Hernia repair			
Patients, No.	12 275	7235	1142
Overall infection	96 (0.8)	53 (0.7)	13 (1.1)
Superficial incisional infection	64 (0.5)	35 (0.5)	8 (0.7)
Deep incisional infection	24 (0.2)	12 (0.2)	4 (0.4)
Organ incisional infection	8 (0.1)	6 (0.1)	1 (0.1)
Hysterectomy			
Patients, No.	2916	2385	225
Overall infection	74 (2.5)	77 (3.2)	4 (1.8)
Superficial incisional infection	18 (0.6)	13 (0.5)	2 (0.9)
Deep incisional infection	12 (0.4)	22 (0.9)	0
Organ incisional infection	44 (1.5)	42 (1.8)	2 (0.9)
Cardiac surgery			
Patients, No.	3372	10 492	3468
Overall infection	188 (5.6)	572 (5.5)	214 (6.2)
Superficial incisional infection	68 (2.0)	184 (1.8)	71 (2.0)
Deep incisional infection	57 (1.7)	137 (1.3)	58 (1.7)
Organ incisional infection	63 (1.9)	248 (2.4)	83 (2.4)
Laminectomy			
Patients, No.	2064	3772	750
Overall infection	25 (1.2)	38 (1.0)	10 (1.3)
Superficial incisional infection	14 (0.7)	14 (0.4)	6 (0.8)
Deep incisional infection	5 (0.2)	9 (0.2)	2 (0.3)
Organ incisional infection	6 (0.3)	15 (0.4)	2 (0.3)
Spondylodesis			
Patients, No.	627	1636	522
Overall infection	16 (2.6)	53 (3.2)	16 (3.1)
Superficial incisional infection	2 (0.3)	13 (0.8)	4 (0.8)
Deep incisional infection	4 (0.6)	3 (0.2)	2 (0.4)
Organ incisional infection	9 (1.4)	37 (2.3)	9 (1.7)
Gastric bypass			
Patients, No.	2283	2993	634
Overall infection	69 (3.0)	126 (4.2)	40 (6.3)
Superficial incisional infection	31 (1.4)	46 (1.5)	27 (4.3)
Deep incisional infection	6 (0.3)	7 (0.2)	1 (0.2)
Organ incisional infection	32 (1.4)	73 (2.4)	12 (1.9)
Total hip arthroplasty			
Patients, No.	19 903	43 465	8164
Overall infection	229 (1.2)	583 (1.3)	119 (1.5)
Superficial incisional infection	67 (0.3)	143 (0.3)	28 (0.3)
Deep incisional infection	30 (0.2)	74 (0.2)	21 (0.3)
Organ incisional infection	130 (0.7)	361 (0.8)	69 (0.8)
Total knee arthroplasty			
Patients, No.	13 885	30 768	6989
Overall infection	146 (1.1)	289 (0.9)	88 (1.3)
Superficial incisional infection	47 (0.3)	93 (0.3)	32 (0.5)
Deep incisional infection	13 (0.1)	36 (0.1)	12 (0.2)
Organ incisional infection	85 (0.6)	158 (0.5)	44 (0.6)

Abbreviations: SAP, surgical antimicrobial prophylaxis; SSI, surgical site infection.

In the adjusted multilevel model, cefuroxime SAP administered 0 to 30 minutes prior to incision in 77 228 patients (34.7%) was associated with a 15% lower SSI rate (adjusted odds ratio [aOR], 0.85; 95% CI, 0.78-0.93; *P* < .001) compared with SAP administration within 61 to 120 minutes prior to incision in 27 207 patients (12.2%). SAP administration 31 to 60 minutes prior to incision (108 004 patients, 53.1%) was also associated with a significantly lower SSI rate (aOR, 0.91; 95% CI, 0.84-0.98; *P* = .01) compared with SAP administration 61 to 120 minutes prior to incision.

Covariables independently associated with a higher SSI rate were (1) an ASA score of 3 to 5 compared with an ASA score of 1 to 2 (aOR, 1.71; 95% CI, 1.60-1.83; *P* < .001), (2) a hospital bed size of more than 500 beds compared with fewer than 200 beds (aOR, 1.30; 95% CI, 1.20-1.42; *P* < .001), (3) procedures longer than standard operation time (aOR, 1.65; 95% CI, 1.55-1.77; *P* < .001), and (4) being 40 years of age or older (aOR, 1.12; 95% CI, 1.05-1.20; *P* < .001). In contrast, sex (aOR for female, 0.79; 95% CI, 0.74-0.84; *P* < .001) and increasing year of data collection (aOR per year, 0.96; 95% CI, 0.96-0.96; *P* < .001) were significantly associated with a lower risk of SSI (Table 3). In eTable 3 in Supplement 1, an additional analysis that included the variable implant (used for arthroplasty and heart surgery with cerclage or valve replacement) and additional categories for age and ASA yielded similar results as the main analysis.

**Table 3.** Fully Adjusted Mixed Effects Logistic Regression Models With Surgical Site Infection as the Dependent Variable^a^

Variable	aOR (95% CI)	*P* value
Timing of cefuroxime surgical antimicrobial prophylaxis administration prior to incision		
0-30 min	0.85 (0.78-0.93)	<.001
31-60 min	0.91 (0.84-0.98)	.01
61-120 min	1 [Reference]	NA
Sex		
Female	0.79 (0.74-0.84)	<.001
Male	1 [Reference]	NA
Age		
≥40 y	1.12 (1.05-1.2)	.001
<40 y	1 [Reference]	NA
American Society of Anesthesiologists score		
3-5	1.71 (1.61-1.83)	<.001
1-2	1 [Reference]	NA
Wound contamination class		
Class II (clean-contaminated)	1.38 (0.9-2.11)	.14
Class I (clean)	1 [Reference]	NA
Duration of procedure exceeded standard time		
Yes	1.65 (1.55-1.77)	<.001
No	1 [Reference]	NA
Hospital bed size, No. of beds		
<200	1 [Reference]	NA
200-500	1.12 (1.05-1.2)	.001
>500	1.3 (1.2-1.42)	<.001
Year (per year increase)	0.96 (0.96-0.96)	<.001

Abbreviations: aOR, adjusted odds ratio; NA, not applicable.

^a^ Procedure type was added as random effect with only complete cases (221 514 of 222 439 patients).

Of the 162 796 evaluable patients in the subgroup analysis, a total of 117 348 patients (72%) received cefuroxime SAP within a window of 30 to 55 minutes prior to incision (as proxy for administration in the preoperating room) compared with 45 448 (28%) patients within a window of 10 to 25 minutes prior to incision (as proxy for application in the operating room). The latter was associated with an 11% lower SSI rate (aOR, 0.89; 95% CI, 0.82-0.97; *P* = .009) (eTable 4 in Supplement 1) compared with administration 30 to 55 minutes prior to incision.

## Discussion

### Principal Findings

The results of this large prospective cohort study showed a statistically significant 15% lower odds of SSI when cefuroxime SAP was administered closer to the time of incision (ie, at 0-30 minutes vs 61-120 minutes). Within the 60-minute time window before incision, a subgroup analysis showed that SAP administration between 10 and 25 minutes before incision was associated with 11% lower odds of SSI than at 30 to 55 minutes, suggesting that administration of cefuroxime SAP should be within 1 hour before incision, ideally within 10 to 25 minutes before incision.

Our results were in contrast to the current largest RCT^14^ on the subject, which was unable to demonstrate a difference. We included a large number of patients in our study (222 439 in our study vs 5580 in the RCT^14^), which could have identified smaller effect sizes, and we applied a hierarchical model that takes into account the variability due to different procedure types. The RCT^14^ may have been confounded, as a higher dose of SAP for patients with a body weight above 80 kg was introduced during the study period, which could have interacted with the timing windows. Nonetheless, a recent study^20^ by our group demonstrated that doubling the dose of cefuroxime in most surgical procedures does not decrease SSI rates. In addition, the RCT^14^ was stratified by center and may have been confounded by differences between the 2 participating centers (eg, different case mix as well as experience and skills of surgical staff).

Our main results indicating administration of SAP close to the time incision are supported by pharmacokinetic data showing mean serum concentration peaks already 3 minutes after intravenous cefuroxime administration and a half-life of 30 minutes.^27^ From an organizational perspective, it has been shown that the completion of SAP administration was greater than 98%, and, therefore, very reliable if given immediately after entering the operating room.^15^

Although our primary exposure variable was the exact timing of SAP administration, our data confirm findings from many previous studies^4,5,6,20,21,28^ that other confounders are significantly associated with SSI risk: higher ASA score, clean-contaminated wound classes (vs clean), and surgical procedures exceeding standard time were associated with an increased risk, as shown elsewhere. The duration of the surgical procedure can be impacted by intraoperative complications that may further affect the healing process and increase the risk for SSI. The overall decrease in SSI over the course of the study could imply that systematic SSI prevention measures were put into place over time in some of the participating centers. Alternatively, an increase in smaller participating hospitals, may have led to a dilution of the high infection rate of the tertiary centers.^29^ By excluding wound contamination classes III and IV and emergency surgery, we limited potential bias by preexisiting infections, antimicrobial therapies, and potentially disrupted optimal surgical preparation. Minor differences in ASA reporting may have occurred over time, as some hospitals may have introduced the modified 2014 ASA classifications.^22^ We do not think that this change would have introduced a relevant bias to the main results. No unusual clusters of exogenous SSI were observed during the study period.

### External and Internal Validity

We consider the external validity of our study to be high for countries with similar health systems, because most hospitals throughout Switzerland participated, ranging from smaller institutions to all university hospitals. High external validity is further corroborated by the large sample size, the long duration of the study, postdischarge surveillance at 30 days (or 1 year for arthroplasty surgery), relatively small (9.1%) loss to follow-up, and the avoidance of too strict inclusion and exclusion criteria. Generalizability may be impacted, however, because this study is a single-country study. Because more than 50% of the participants underwent an arthroplasty procedure, applicability to other surgical procedures may be limited.

For the crude SSI rate (Figure 2), we were able to stratify the timing window of SAP by 10-minute intervals, which is difficult to accomplish in a clinical trial setting with a limited number of patients and treatment groups. The multilevel analysis with clustering at the intervention level also allowed us to control for potential variation in SSI rate between different surgical procedures. We adjusted hospital bed size and individual factors (age, ASA score, and duration of surgery) to reduce confounding bias. The internal validity was high, as our study involved routine on-site monitoring of the data.

### Implications

Our results suggest that cefuroxime SAP should be administered within 60 minutes prior to incision to reduce SSI risk. The subgroup analysis suggests a window ideally between 10 and 25 minutes before incision, which was associated with the lowest risk of SSI.

In consideration of the very large sample size of the present cohort study, further studies must involve multiple, international cohort data. Furthermore, the association found here should be verified for agents other than cefuroxime, with different half-lives, volume of distribution, and tissue levels.

### Limitations

The main limitation of the study was that variables were predefined by the SSI surveillance program. Important comorbidities and characteristics of patients, as well as periprocedural variables, such as diabetes and perioperative glucose control, immunosuppressive status, radiation status, preoperative infection status at remote sites, preoperative decolonization status, perioperative skin antisepsis measures, smoking (including preoperative cessation), nutritional status, intraoperative temperature, or oxygen measurements were not available.^20^ All of these variables may impact wound healing and may represent a potential source of residual confounding. In addition, information on intraoperative and postoperative redosing and extended prophylaxis was limited and excluded from the study. Because we only had information on the cefuroxime dosing regimen for 52.0% of the cohort and information on body-mass index for 64.0% of patients, we also excluded these 2 potentially important confounding variables. Additional bias may have been introduced because patients may not have been eligible to receive cefuroxime as a prophylactic agent at all institutions. Unfortunately, we were not able to review the institutional policies and to estimate the size and direction of this bias.

Next, there may have been confounding by indication: an SAP administration at 61 to 120 minutes before incision may be used for more complex surgeries that lead to higher infection rate, potentially overestimating the significant association of late SAP administration (ie, 0-30 minutes before incision). Situations that may require an SAP administration between 61 and 120 minutes before incision include preparing the operating room for patients with obesity, inexperienced surgical teams that are not well coordinated or need more time in the operating room before starting surgery, or when junior surgeons have to wait for the arrival of their supervising surgeon.

Of importance, we cannot exclude residual confounding despite adjustment of the results. Multiple variables that intrinsically had lower risk of SSI (female sex, younger age, and lower ASA score) were overrepresented in the 0 to 30 minute group, relative to the other timing groups. Also, the lower rate of SSI associated with a smaller hospital bed size is of concern, since surgical volume is associated with lower SSI rates.^30^ However, we think that this bias is rather small, underlined by the fact that the results were almost unchanged even in our additional analysis (eTable 3 in Supplement 1).

## Conclusions

In this cohort study of 222 439 patients who underwent 1 of 11 major surgical procedures, administration of cefuroxime prophylaxis closer to the time of incision was associated with significantly lower odds of SSI. These findings suggest that cefuroxime surgical prophylaxis should be administrated within 60 minutes prior to incision, ideally within 10 to 25 minutes.

## Notes

Supplement 1. **eFigure. **Administration of Surgical Antimicrobial Prophylaxis Relative to Timing Before Incision (Histogram) **eTable 1.** Baseline and Procedural Characteristics of Patients Included and Lost to Follow-Up **eTable 2.** Summary of the Leading Microorganism Detected in 3381 of 222 439 Cases (1.52%) With Recorded Etiology by SAP Timing Group **eTable 3.** Fully Adjusted Mixed Effects Logistic Regression Models With Surgical Site Infection as the Dependent Variable, Including the Variable *Implant* and More Categories for Age and ASA **eTable 4.** Fully Adjusted Mixed Effects Logistic Regression Models With Surgical Site Infection as the Dependent Variable for the Subgroup Preparatory Room (55-30 Minutes Prior to Incision) vs Operatory Room (25-10 Minutes Prior to Incision)

Supplement 2.
Nonauthor Collaborators

Supplement 3.
Data Sharing Statement
