# Supplementary material for: Timing of Cefuroxime Surgical Antimicrobial Prophylaxis and Its Association With Surgical Site Infections
Source: JAMA Netw Open. 2023 Jun 8;6(6):e2317370. doi: 10.1001/jamanetworkopen.2023.17370 (PMC10251212; doi:10.1001/jamanetworkopen.2023.17370)
Supplement: Supplement 1. — eFigure. Administration of Surgical Antimicrobial Prophylaxis Relative to Timing Before Incision (Histogram) eTable 1. Baseline and Procedural Characteristics of Patients Included and Lost to Follow-Up eTable 2. Summary of the Leading Microorganism Detected in 3381 of 222 439 Cases (1.52%) With Recorded Etiology by SAP Timing Group eTable 3. Fully Adjusted Mixed Effects Logistic Regression Models With Surgical Site Infection as the Dependent Variable, Including the Variable Implant and More Categories for Age and ASA eTable 4. Fully Adjusted Mixed Effects Logistic Regression Models With Surgical Site Infection as the Dependent Variable for the Subgroup Preparatory Room (55-30 Minutes Prior to Incision) vs Operatory Room (25-10 Minutes Prior to Incision) [file jamanetwopen-e2317370-s001.pdf]

# Supplemental Online Content

Sommerstein R, Troillet N, Harbarth S, et al; Swissnoso group. Timing of cefuroxime surgical antimicrobial prophylaxis and its association with surgical site infections. *JAMA Netw Open*. 2023;6(6):e2317370. doi:10.1001/jamanetworkopen.2023.17370

**eFigure.** Administration of Surgical Antimicrobial Prophylaxis Relative to Timing Before Incision (Histogram)
**eTable 1.** Baseline and Procedural Characteristics of Patients Included and Lost To Follow-up
**eTable 2.** Summary of the Leading Microorganism Detected in 3381 of 222 439 Cases (1.52 %) With Recorded Etiology by SAP Timing Group
**eTable 3.** Fully Adjusted Mixed Effects Logistic Regression Models With Surgical Site Infection as the Dependent Variable, Including the Variable *Implant* and More Categories for Age and ASA
**eTable 4.** Fully Adjusted Mixed Effects Logistic Regression Models With Surgical Site Infection as the Dependent Variable for the Subgroup Preparatory Room (55-30 Minutes Prior to Incision) vs Operatory Room (25-10 Minutes Prior to Incision)

This supplemental material has been provided by the authors to give readers additional information about their work.

© 2023 Sommerstein R et al. *JAMA Network Open.*

**Supplementary Material**

**eFigure**

Administration of surgical antimicrobial prophylaxis relative to timing before incision (histogram).

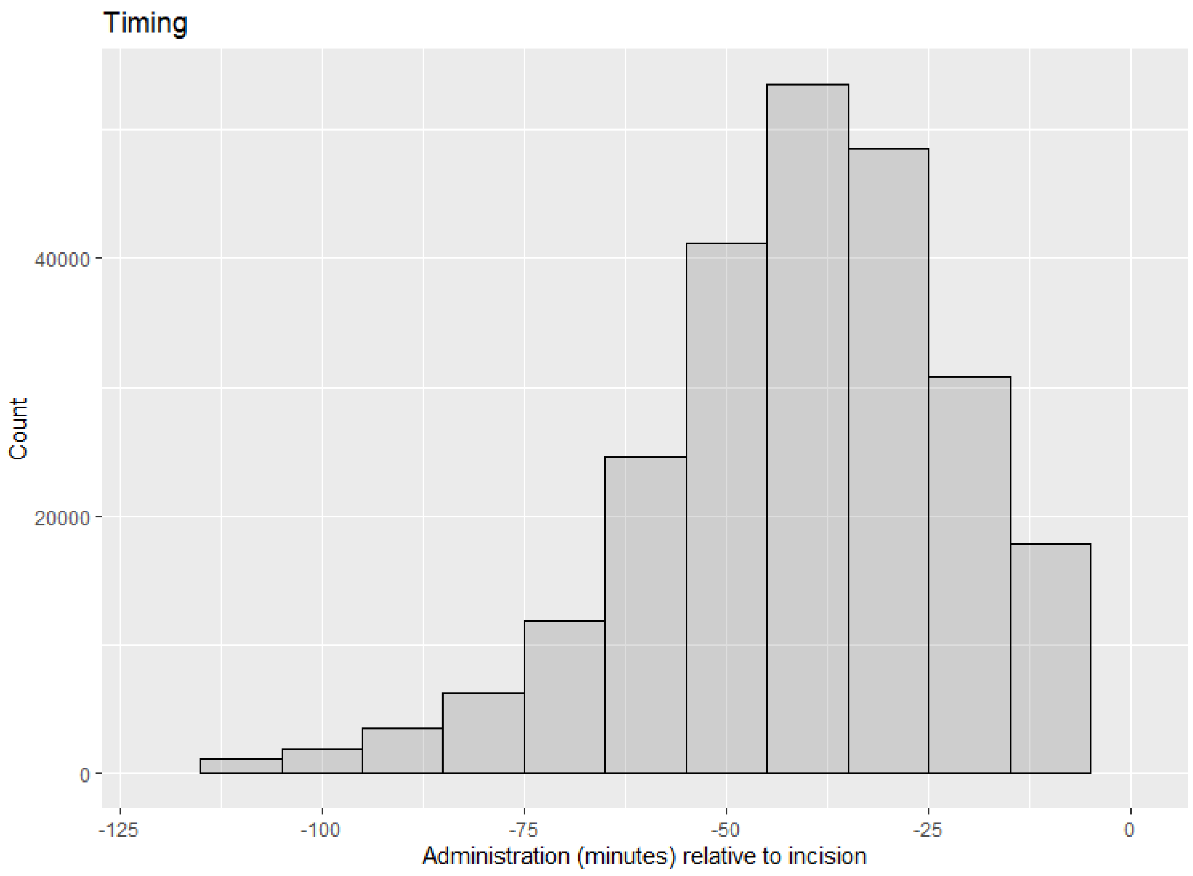


© 2023 Sommerstein R et al. *JAMA Network Open.*

## eTable 1

## Baseline and Procedural Characteristics of Patients Included and Lost to Follow-up

| Characteristic | Included Patients, No (%) | Lost to Follow-up Patients, No (%) | |
|---|---|---|---|
| n | 222439 | 22208 | |
| Age, median (IQR), y | 65.69 [53.87, 74.20] | 63.02 [50.54, 73.13] | <0.001 |
| Sex = f | 118392 (53.2) | 12509 (56.3) | <0.001 |
| ASA scores | | | <0.001 |
| 1, 2 | 154749 (69.6) | 15074 (67.9) | |
| 3-5 | 66765 (30.0) | 7008 (31.6) | |
| NA | 925 ( 0.4) | 126 ( 0.6) | |
| Addition of Metronidazole as second SAP | 19397 (8.7) | 1342 (6.0) | <0.001 |
| Intervention Type | | | <0.001 |
| Cesarean Section | 13426 ( 6.0) | 1939 ( 8.7) | |
| Cholecystectomy | 9831 ( 4.4) | 1047 ( 4.7) | |
| Colon surgery | 17217 ( 7.7) | 1068 ( 4.8) | |
| Hernia repair | 20652 ( 9.3) | 1332 ( 6.0) | |
| Hysterectomy | 5526 ( 2.5) | 789 ( 3.6) | |
| Cardiac surgery | 17332 ( 7.8) | 2490 (11.2) | |
| Laminectomy | 6586 ( 3.0) | 370 ( 1.7) | |
| Spondylodesis | 2785 ( 1.3) | 260 ( 1.2) | |
| Gastric bypass surgery | 5910 ( 2.7) | 560 ( 2.5) | |
| Total Hip Prosthesis | 71532 (32.2) | 6656 (30.0) | |
| Total Knee Prosthesis | 51642 (23.2) | 5697 (25.7) | |
| Wound contamination class = clean-contaminated (vs. clean) | 52732 (23.7) | 5498 (24.8) | <0.001 |
| Surgery exceeding standard time = yes | 31807 (14.3) | 3106 (14.0) | 0.21 |
| Year (median [IQR]) | 2015.00 [2013.00, 2018.00] | 2015.00 [2012.00, 2018.00] | <0.001 |
| Hospital size (beds) | | | <0.001 |
| <200 | 126947 (57.1) | 13325 (60.0) | |
| 200-499 | 67516 (30.4) | 5782 (26.0) | |
| 500+ | 27976 (12.6) | 3101 (14.0) | |

Characteristics showed (among others), that patients lost to follow up were younger, more often female, and underwent more frequently Cesearen section.

© 2023 Sommerstein R et al. *JAMA Network Open.*

## eTable 2

## Summary of the Leading Microorganism Detected in 3381 of 222 439 Cases (1.52 %) With Recorded Etiology by SAP Timing Group

| | Administration of SAP prior to incision | | |
|---|---|---|---|
| **Microbiologic etiology** | **0-30 minutes Patients, No (%)** | **31-60 minutes Patients, No (%)** | **61-120 minutes Patients, No (%)** |
| | n=1 103 | n=1 652 | n=626 |
| Escherichia coli | 103 (17.0) | 258 (21.7) | 102 (21.9) |
| Methicillin-susceptible Staphyloccocus aureus | 109 (18.0) | 185 (15.6) | 64 (13.7) |
| Coagulase-negative Staphylococcus | 91 (15.0) | 172 (14.5) | 64 (13.7) |
| Enterococci (VRE or non VRE) | 29 ( 4.8) | 82 ( 6.9) | 39 ( 8.4) |
| ESBL-producing Escherichia coli | 17 ( 2.8) | 33 ( 2.8) | 21 ( 4.5) |
| Alpha-haemolytic Streptococcus | 27 ( 4.5) | 46 ( 3.9) | 16 ( 3.4) |
| Enterococcus faecium (non VRE) | 12 ( 2.0) | 26 ( 2.2) | 15 ( 3.2) |
| Enterobacter aerogenes / cloacae | 22 ( 3.6) | 38 ( 3.2) | 15 ( 3.2) |
| Carbapenemase-producing Enterobacter aerogenes / cloacae | 20 ( 3.3) | 22 ( 1.9) | 14 ( 3.0) |
| Enterococcus faecalis (non VRE) | 21 ( 3.5) | 41 ( 3.4) | 13 ( 2.8) |
| Pseudomonas aeruginosa | 20 ( 3.3) | 38 ( 3.2) | 12 ( 2.6) |
| Cutibacterium acnes | 16 ( 2.6) | 37 ( 3.1) | 10 ( 2.1) |
| Proteus mirabilis, vulgaris | 7 ( 1.2) | 14 ( 1.2) | 9 ( 1.9) |
| Bacteroïdes sp. | 13 ( 2.1) | 23 ( 1.9) | 8 ( 1.7) |
| Candida albicans | 8 ( 1.3) | 8 ( 0.7) | 8 ( 1.7) |
| Streptococcus agalactiae | 12 ( 2.0) | 28 ( 2.4) | 7 ( 1.5) |
| Klebsiella pneumoniae, oxytoca, variicola | 16 ( 2.6) | 24 ( 2.0) | 6 ( 1.3) |
| Other streptococcus | 14 ( 2.3) | 32 ( 2.7) | 6 ( 1.3) |
| Methicillin-resistent Staphyloccocus aureus (MRSA) | 8 ( 1.3) | 5 ( 0.4) | 5 ( 1.1) |
| Other Gram positive | 4 ( 0.7) | 9 ( 0.8) | 4 ( 0.9) |
| Gemella sp. | 6 ( 1.0) | 10 ( 0.8) | 4 ( 0.9) |
| Serratia marcescens | 3 ( 0.5) | 5 ( 0.4) | 3 ( 0.6) |
| Clostridium species | 2 ( 0.3) | 0 ( 0.0) | 3 ( 0.6) |
| Corynebacterium sp | 2 ( 0.3) | 5 ( 0.4) | 2 ( 0.4) |
| ESBL-producing Klebsiella sp. | 1 ( 0.2) | 3 ( 0.3) | 2 ( 0.4) |
| Prevotella sp. | 3 ( 0.5) | 3 ( 0.3) | 2 ( 0.4) |
| Candida glabrata | 1 ( 0.2) | 2 ( 0.2) | 2 ( 0.4) |
| Streptococcus pyogenes | 1 ( 0.2) | 3 ( 0.3) | 2 ( 0.4) |
| Gut Flora | 1 ( 0.2) | 6 ( 0.5) | 2 ( 0.4) |
| VRE | 1 ( 0.2) | 4 ( 0.3) | 1 ( 0.2) |
| Bacillus sp. | 0 ( 0.0) | 3 ( 0.3) | 1 ( 0.2) |
| Acinetobacter sp. | 1 ( 0.2) | 3 ( 0.3) | 1 ( 0.2) |
| Haemophilus sp | 3 ( 0.5) | 1 ( 0.1) | 1 ( 0.2) |
| Other Gram negatives | 1 ( 0.2) | 0 ( 0.0) | 1 ( 0.2) |
| Veillonellae | 0 ( 0.0) | 1 ( 0.1) | 1 ( 0.2) |

© 2023 Sommerstein R et al. *JAMA Network Open.*

| | | | |
|---|---|---|---|
| Carbapenemase-producing Escherichia coli | 2 ( 0.3) | 0 ( 0.0) | 0 ( 0.0) |
| ESBL-producing Enterobacter aerogenes / cloacae | 0 ( 0.0) | 1 ( 0.1) | 0 ( 0.0) |
| Stenotrophomonas maltophilia | 1 ( 0.2) | 0 ( 0.0) | 0 ( 0.0) |
| Streptococcus pneumoniae | 0 ( 0.0) | 2 ( 0.2) | 0 ( 0.0) |
| Clostridium perfringens | 0 ( 0.0) | 1 ( 0.1) | 0 ( 0.0) |
| Peptostreptococcus sp | 0 ( 0.0) | 7 ( 0.6) | 0 ( 0.0) |
| Other Candida sp. | 0 ( 0.0) | 2 ( 0.2) | 0 ( 0.0) |
| Other fungi | 1 ( 0.2) | 2 ( 0.2) | 0 ( 0.0) |
| Non-classifiable | 5 ( 0.8) | 3 ( 0.3) | 0 ( 0.0) |
| Skin Flora | 1 ( 0.2) | 1 ( 0.1) | 0 ( 0.0) |

© 2023 Sommerstein R et al. *JAMA Network Open.*

## eTable 3

**Fully Adjusted Mixed Effects Logistic Regression Models With Surgical Site Infection as the Dependent Variable, Including the Variable *Implant* and More Categories for Age and ASA**

Procedure type was added as random effect. Only complete cases (221 514/222 439)

| **Variable** | aOR | Lower CI | Upper CI | p-value |
|---|---|---|---|---|
| Cefuroxime timing 0-30 minutes (Ref: 61-120 minutes) | 0.85 | 0.78 | 0.93 | <0.001 |
| Cefuroxime timing 31-60 minutes (Ref: 61-120 minutes) | 0.91 | 0.84 | 0.98 | 0.01 |
| ASA Score 2 (Ref = ASA 1) | 1.43 | 1.27 | 1.61 | <0.001 |
| ASA Score 3 (Ref = ASA 1) | 2.40 | 2.11 | 2.73 | <0.001 |
| ASA Score 4 (Ref = ASA 1) | 2.82 | 2.37 | 3.36 | <0.001 |
| ASA Score 5 (Ref = ASA 1) | 2.27 | 0.89 | 5.81 | 0.09 |
| Age group 30-40 years (Ref: 18-20 years) | 1.01 | 0.83 | 1.23 | 0.92 |
| Age group 40-50 years (Ref: 18-20 years) | 1.00 | 0.82 | 1.21 | 0.96 |
| Age group 50-60 years (Ref: 18-20 years) | 0.93 | 0.77 | 1.12 | 0.44 |
| Age group 60-70 years (Ref: 18-20 years) | 0.86 | 0.71 | 1.04 | 0.12 |
| Age group 70-80 years (Ref: 18-20 years) | 0.81 | 0.66 | 0.98 | 0.03 |
| Age group 80-90 years (Ref: 18-20 years) | 0.75 | 0.61 | 0.93 | 0.007 |
| Age group >90 years (Ref: 18-20 years) | 0.78 | 0.52 | 1.17 | 0.23 |
| Wound contamination class: Clean-contaminated (Ref = clean) | 1.40 | 0.89 | 2.19 | 0.14 |
| Implant = yes (Ref= no implant) | 1.13 | 0.91 | 1.39 | 0.27 |
| Duration exceeding standard time (Ref = No) | 1.64 | 1.54 | 1.75 | <0.001 |
| Female Sex (Ref=male) | 0.79 | 0.74 | 0.84 | <0.001 |
| Hospital Size 200-499 beds (Ref= <200 beds) | 1.12 | 1.05 | 1.19 | 0.001 |
| Hospital Size >500 beds (Ref= <200 beds) | 1.27 | 1.17 | 1.39 | <0.001 |
| Year (per year increase) | 0.96 | 0.96 | 0.96 | <0.001 |

Abbreviations

aOR Adjusted odds ratio

ASA American Society of Anesthesiologists

CI Confidence interval

© 2023 Sommerstein R et al. *JAMA Network Open.*

**eTable 4**

**Fully Adjusted Mixed Effects Logistic Regression Models With Surgical Site Infection as the Dependent Variable for the Subgroup: Preparatory Room (55-30 Minutes Prior to Incision) vs Operatory Room (25-10 Minutes Prior to Incision)**

Procedure type was added as random effect. Only complete cases (162 129/162 796)

| Variable | aOR and 95% CI | p-value |
|---|---|---|
| Cefuroxime timing 25-10 minutes (Ref: 55-30 minutes) | 0.89 (0.82-0.97) | 0.008 |
| Female Sex (Ref=male) | 0.79 (0.73-0.85) | <0.001 |
| Age ≥40 years (Ref = <40 years) | 0.92 (0.79-1.07) | 0.26 |
| ASA Score 3-5 (Ref = ASA 1 / 2) | 1.61 (1.49-1.75) | <0.001 |
| Wound contamination class: Clean-contaminated (Ref = clean) | 1.38 (0.82-2.31) | 0.23 |
| Duration exceeding standard time (Ref = No) | 1.71 (1.58-1.86) | <0.001 |
| Hospital Size (Ref= <200 beds) | | |
| 200-499 beds | 1.06 (0.97-1.15) | 0.19 |
| 500+ beds | 1.37 (1.24-1.52) | <0.001 |
| Year (per year increase) | 0.96 (0.96-0.96) | <0.001 |

Abbreviations

aOR Adjusted odds ratio

ASA American Society of Anesthesiologists

CI Confidence interval

© 2023 Sommerstein R et al. *JAMA Network Open.*
