# Supplementary material for: Timing of Cefuroxime Surgical Antimicrobial Prophylaxis and Its Association With Surgical Site Infections
Source: JAMA Netw Open. 2023 Jun 8;6(6):e2317370. doi: 10.1001/jamanetworkopen.2023.17370 (PMC10251212; doi:10.1001/jamanetworkopen.2023.17370)
Supplement: Supplement 3. — Data Sharing Statement [file jamanetwopen-e2317370-s003.pdf]

Data Sharing Statement

Sommerstein. Timing of Cefuroxime Surgical Antimicrobial Prophylaxis and Its Association With Surgical Site Infections. *JAMA Netw Open*. Published June 08, 2023. doi:10.1001/jamanetworkopen.2023.17370

Data
Data available: No

Additional Information
Explanation for why data not available: Data are available upon reasonable request. For the process we refer to the Swissnoso data and publication regulations: https://swissnoso.ch/forschung-entwicklung/reglemente
